# Supplementary material for: Integrative functional genomics decodes herpes simplex virus 1
Source: Nat Commun. 2020 Apr 27;11:2038. doi: 10.1038/s41467-020-15992-5 (PMC7184758; doi:10.1038/s41467-020-15992-5)
Supplement: Supplementary file 1 — Supplementary Information [file 41467_2020_15992_MOESM1_ESM.pdf]

Integrative functional genomics decodes herpes simplex virus 1

Whisnant & Juerges et al. 2020

Supplementary Figures

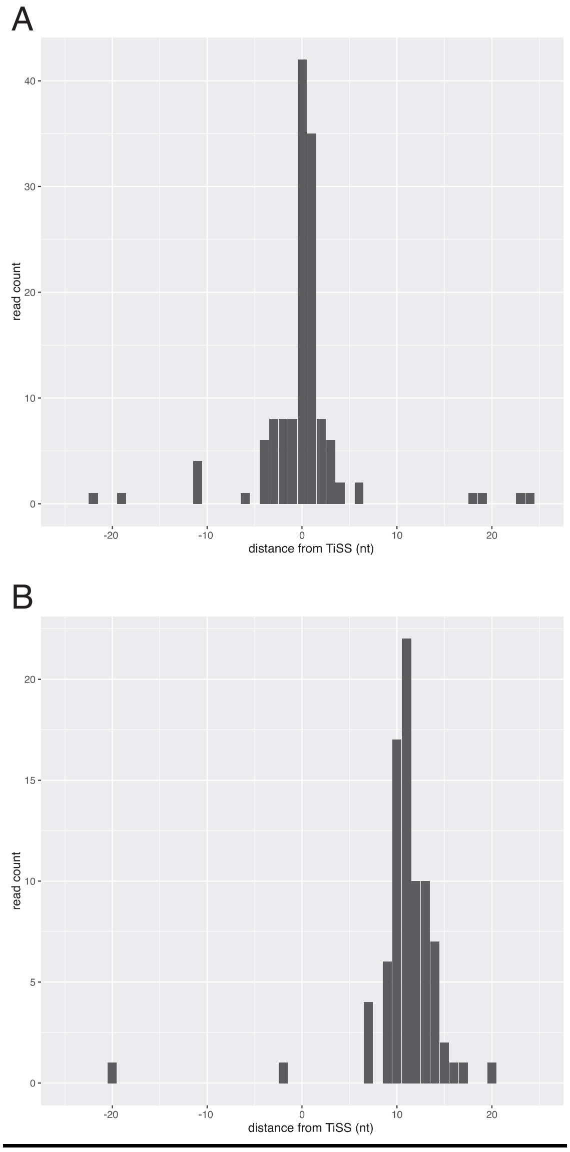

Supplementary Figure 1. Transcription start sites of PacBio and MinION

(**A**) Distance of transcription start sites (TiSS) identified by PacBio (*1*) to the TiSS positions obtained by both cRNA-seq and dRNA-seq is shown in relation to read counts. This confirmed TiSS identified by cRNA-seq and dRNA-seq at single nucleotide resolution. (**B**) Same as for (A) but for TiSS obtained from MinION sequencing data (*2*). The 89 TiSS called by MinION generally lacked 7-18 nucleotides (nt) at the 5' end for technical limitations of the MinION direct RNA sequencing method. After correcting for this, the manually curated MinION data confirmed many of the TiSS we identified.

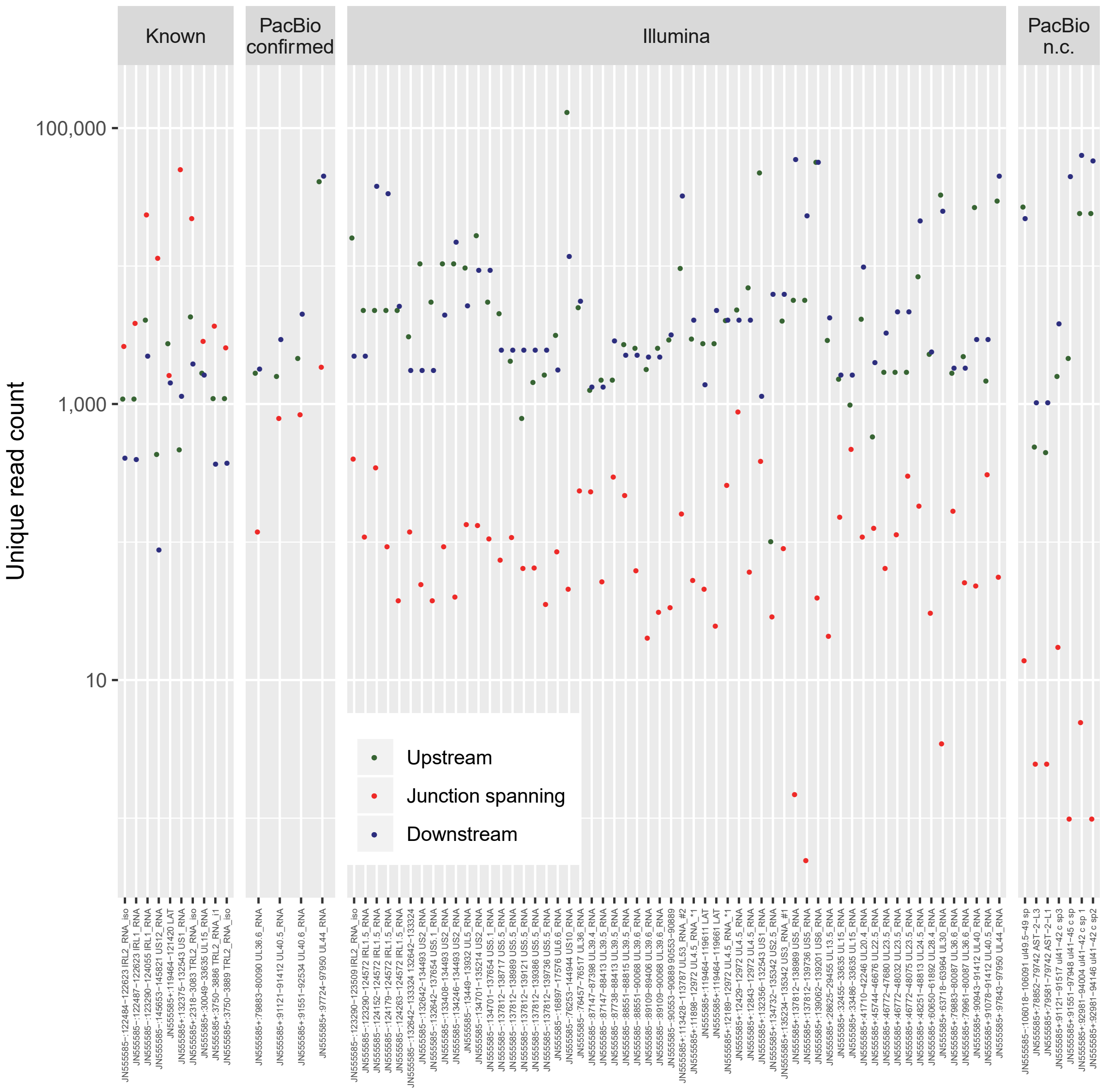

Supplementary Figure 2. Identification of splicing events in the HSV-1 transcriptome

The mapped reads of the 4sU-seq and total RNA-seq experiment were used to examine splicing in the HSV-1 genome. Shown are the locations of known and putative splicing events in the HSV-1 transcriptome as well as one representative transcript for each of them. The putative splicing events are separated into three groups. The ones that were called by PacBio, which we could confirm to be present at reasonable expression levels (PacBio confirmed), the ones that were called by PacBio but did not occur at reasonable levels in our data (PacBio non-confirmed; PacBio n.c.) and the ones that were only identified in our data, but did not occur at reasonable levels (Illumina). For each splice junction the number of unique reads spanning it (red) is depicted as well as the non-spanning reads upstream (green) and downstream (blue). Besides the 8 known splicing events and a NAGNAG event in the ICP0 mRNA, Illumina sequencing confirmed 4 splicing events identified by PacBio (*1*). Furthermore, screening our RNA-seq and 4sU-seq data for splicing events with at least 10 nt

exon-spanning uniquely mapping reads identified 58 putative additional splicing events. Reads with mismatches around the splice-site were removed to assure the NAGANG event is not due to bad mapping. However, exon-spanning reads were >10-fold less prevalent than reads mapping to the flanking regions. We therefore, decided not to include them into our reference annotation. Nevertheless, they may explain some of the orphan ORFs. Our data confirmed all 11 splicing events. However, only four of them occurred at relevant levels and were included into our reference annotation. The small and bright dots represent the read counts of two biological samples (n=2). Their mean is indicated as larger dot.

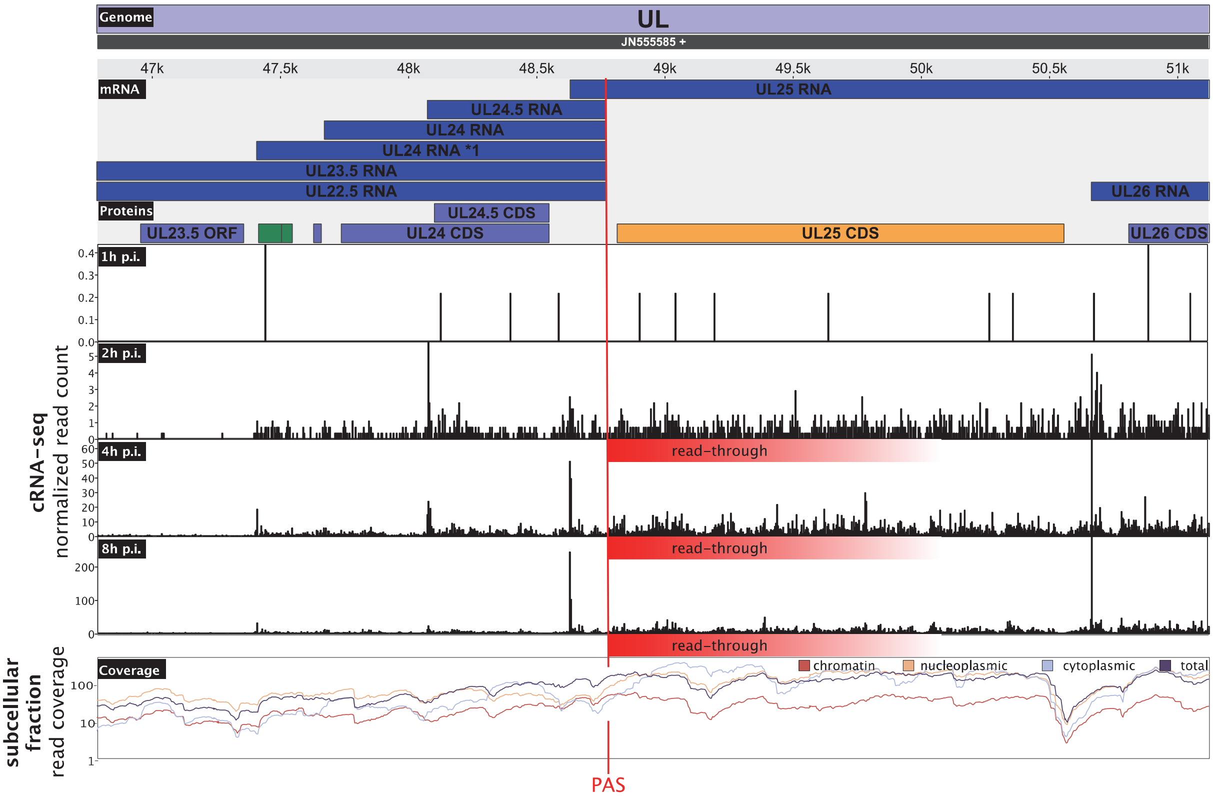

Supplementary Figure 3: Read-through of the UL24 polyadenylation site

Screenshot of our HSV-1 viewer depicting the annotated transcripts (mRNA) and proteins (Proteins) as well as the number of 5' ends of reads for each position of the cRNA-seq dataset. Additionally, the read coverage (Coverage) for chromatin associated, nucleoplasmic, cytoplasmic and total RNA is shown. Cytoplasmic read numbers were notably higher upstream than downstream of the PAS when compared to chromatin-associated dRNA. The read-through (marked in red) of the UL24 polyadenylation site (PAS) that results from transcription of UL25 can be seen as early as 2 h p.i. This confirms previous findings on differential polyadenylation of selected viral genes during productive infection.

A

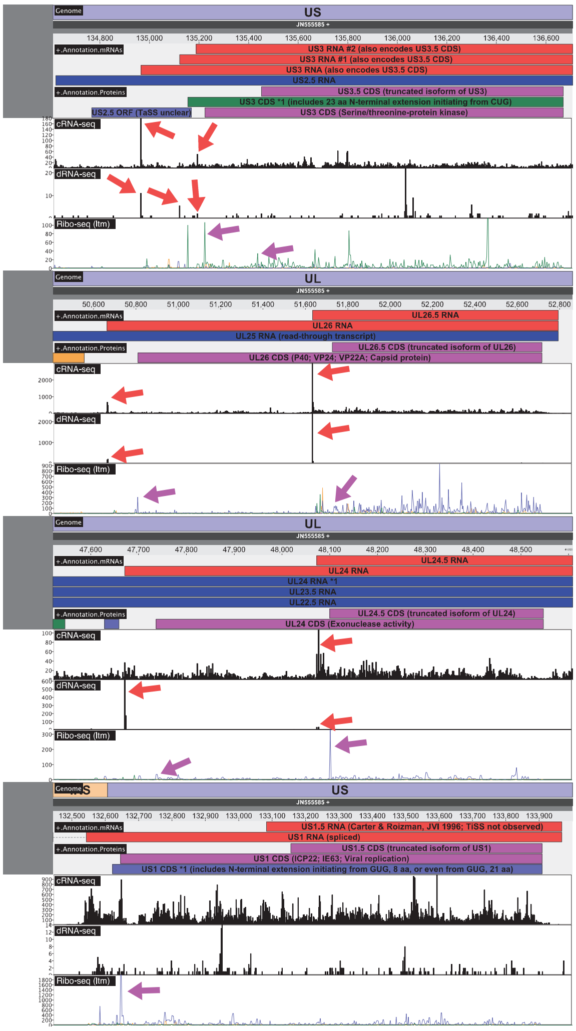

B

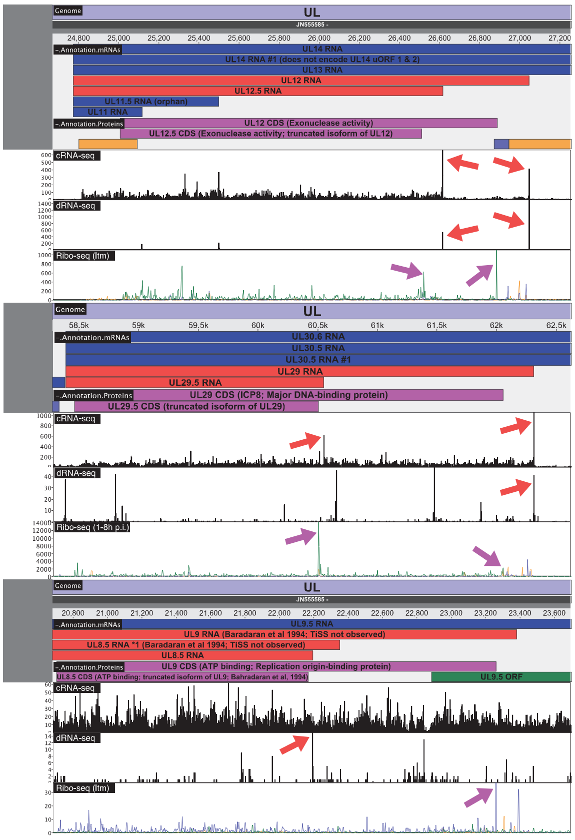

Supplementary Figure 4: Large truncated viral ORFs

Screenshots of our HSV-1 viewer depicting the seven viral open reading frames (ORFs) with N-terminal extended ORFs (NTEs). Alternative transcription start sites (TiSS) downstream of the main TiSS explained translation of 6 of 7 N-terminal truncated ORFs (NTTs). Only for US3.5, we could not identify a corresponding transcript. The NTTs as well the original ORFs are highlighted in pink. The corresponding transcripts for both are highlighted in red. Red arrows in the cRNA-seq and dRNA-seq track point at the specific TiSS validation, if present. Pink arrows point at the translation start site (TaSS) validation if present. The three colored graphs depict the read counts for the three possible ORFs (yellow=1, blue=2, green=3) **(A)** NTTs encoded in the sense strand. **(B)** NTTs encoded in the antisense strand. For UL29/UL29.5 the combined ribosome profiling data for 1 h p.i. up to 8 h p.i. is shown instead of the Lactimidomycin data. This was a pure esthetical decision as the TaSS peak there was too prominent and therefore translation throughout the ORF could not be seen anymore.

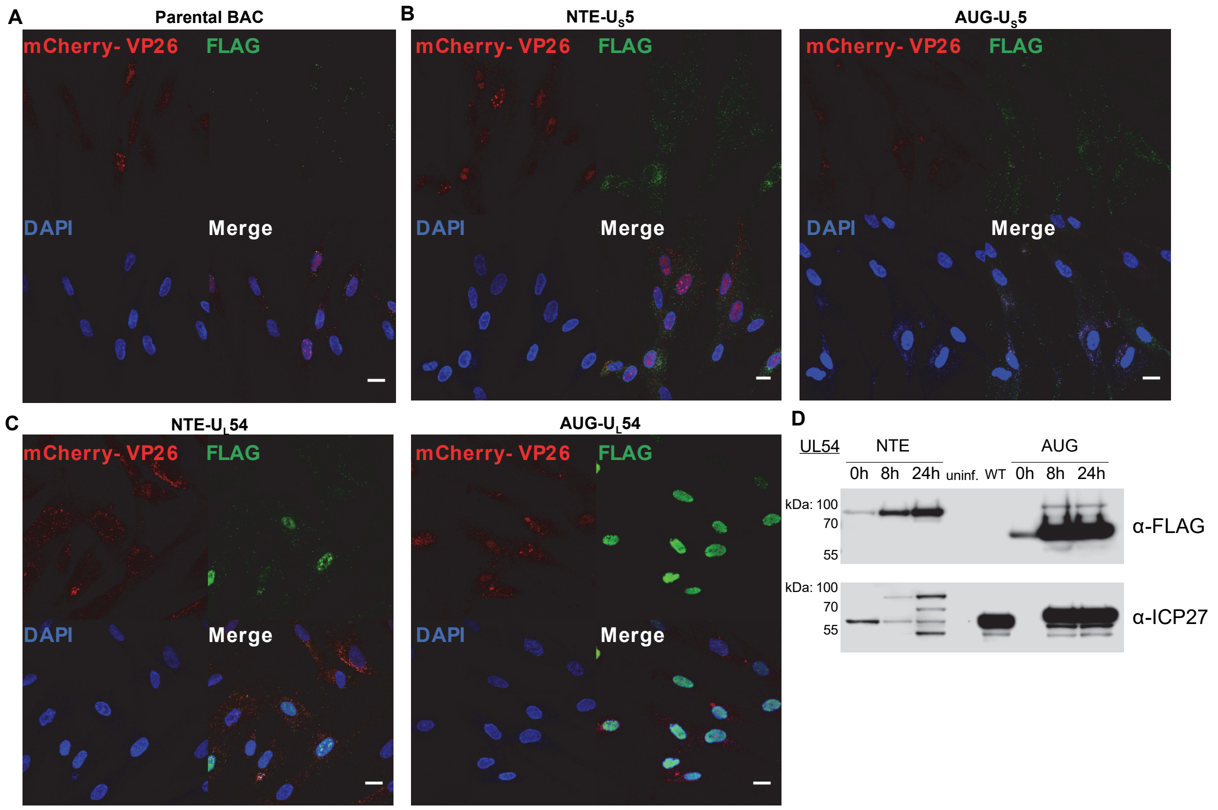

Supplementary Figure 5: Investigation of N-terminal extensions by IF and Western blot
Immunofluorescence (IF) of human foreskin fibroblasts infected with parental BAC-derived VP26-mCherry HSV-1 (**A**) or viruses containing 3X-FLAG-tags inserted upstream of the canonical start codon into the N-terminal extension (NTE) or downstream of it (AUG) for US5 (**B**) and UL54 (**C**). Cell nuclei where stained using DAPI. Scale bars depict 20 microns. No differences in the subcellular localization between the NTE and the canonical protein were observed. (**D**) Western blot for UL54 showing the signal for FLAG as shown in Figure 5 and total UL54/ICP27 levels in the same samples. Source data are provided as a Source Data file.

Virus	Accession number	Amino acid sequence
HSV-1	JN555585	L/MPLLKTPGPVVRGARWLALTVRRM
HSV-2	NC_001798	L/MPLLKTPGPVARGARWLARATRQM
BHV-1	NC_001847	IAGVDRVRLGVRLPFLPQARSRDTTRRSWAPM
FeHV-1	NC_013590	RFLLFRKCIRLANMDRFPRVGLSCCIPTSKGDIDTGDNYKLQSTM
MaHV-1	KT594769	TCLSFQITGSLCM
PRV	NC_006151	MLAMWRWVTKRSRLRRGHAHLGGNKGVRGICSLYLAGLSRGLSRVHAQRSHAATM

Supplementary Figure 6: Prediction of alphaherpesviral US3 N-terminal extensions. Primary peptide sequences for validated (HSV-1, PRV) and predicted (HSV-2, BHV-1, FeHV-1 and MaHV-1) US3 NTEs are depicted from the start codons (canonical or non-canonical) to the annotated US3 start codon ("M" in bold). Hydrophobic resides are indicated in red. Putative nuclear export signals matching the motif [LIVFM]-$X_{2,3}$-[LIVFM]-$X_{2,3}$-[LIVFM]-X-[LIVFM] are highlighted in yellow. The mitochondrial localization signal predicted for PRV (*3*) is underlined.

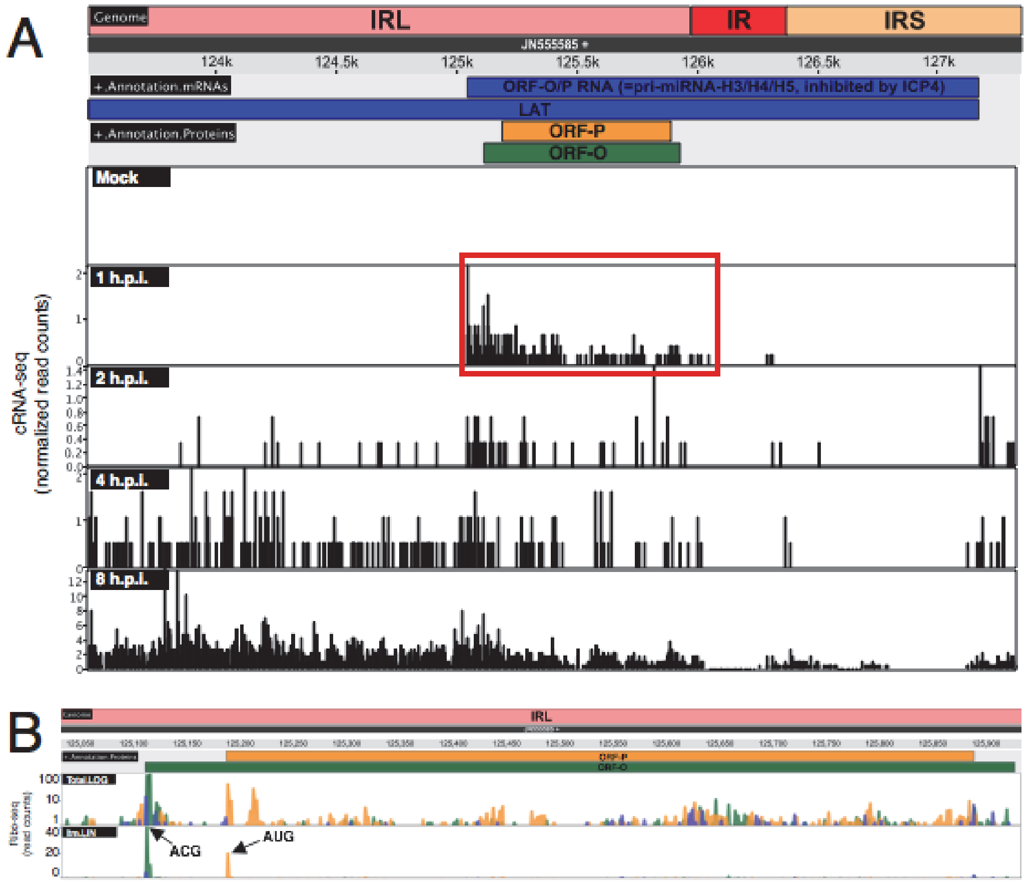

Supplementary Figure 7. Expression of ORF-O and ORF-P

(**A**) Expression kinetics of the ORF-O/ORF-P mRNA depicted by cRNA-seq. While the mature transcript is well expressed at 1 h p.i., transcriptional activity rapidly declines thereafter and is obscured by transcription from upstream genomic regions later on in infection. (**B**) Ribosome profiling (Ribo-seq) data for ORF-O and ORF-P. Combined data from all time points analyzed by standard ribosome profiling (Total log) is shown to account for the overall low translation rates. While translation of ORF-P is well represented, translation of ORF-O is less prominent. While we cannot fully exclude the previously proposed frameshift within ORF-O, a strong translation start site peak obtained by Lactimidomycin treatment 76 nt upstream of the AUG start codon of ORF-P is consistent with ORF-O initiating from an ACG start codon upstream of ORF-P. Colors in the Ribo-seq data depict the three possible open reading rames (yellow=1, blue=2, green=3).

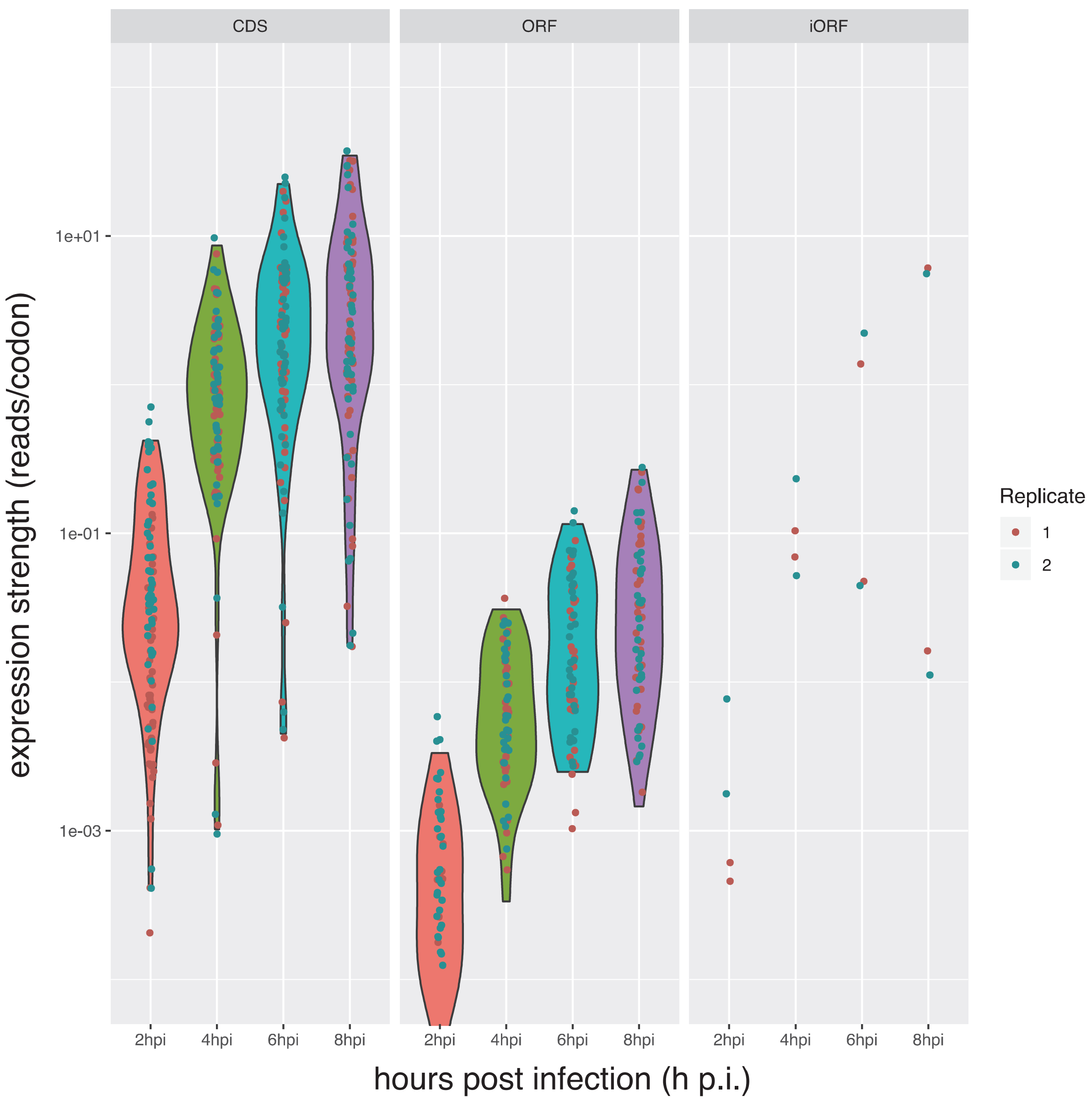

Supplementary Figure 8: Expression strength of all identified large (≥100 aa) ORFs

Expressions strengths of all open reading frames (ORFs) with an amino acid length ≥100 over the course of the infection classified by their respective ORF type. This includes all known large ORFs (CDS) and 41 ORFs and 2 internal ORFS (iORFs). Overlapping ORFs translated in the same frame were excluded. All identified ORFs show lower expressions than the previously identified ones (CDS). Most of the large ORFs are expressed at relatively low levels compared to the known large viral ORFs.

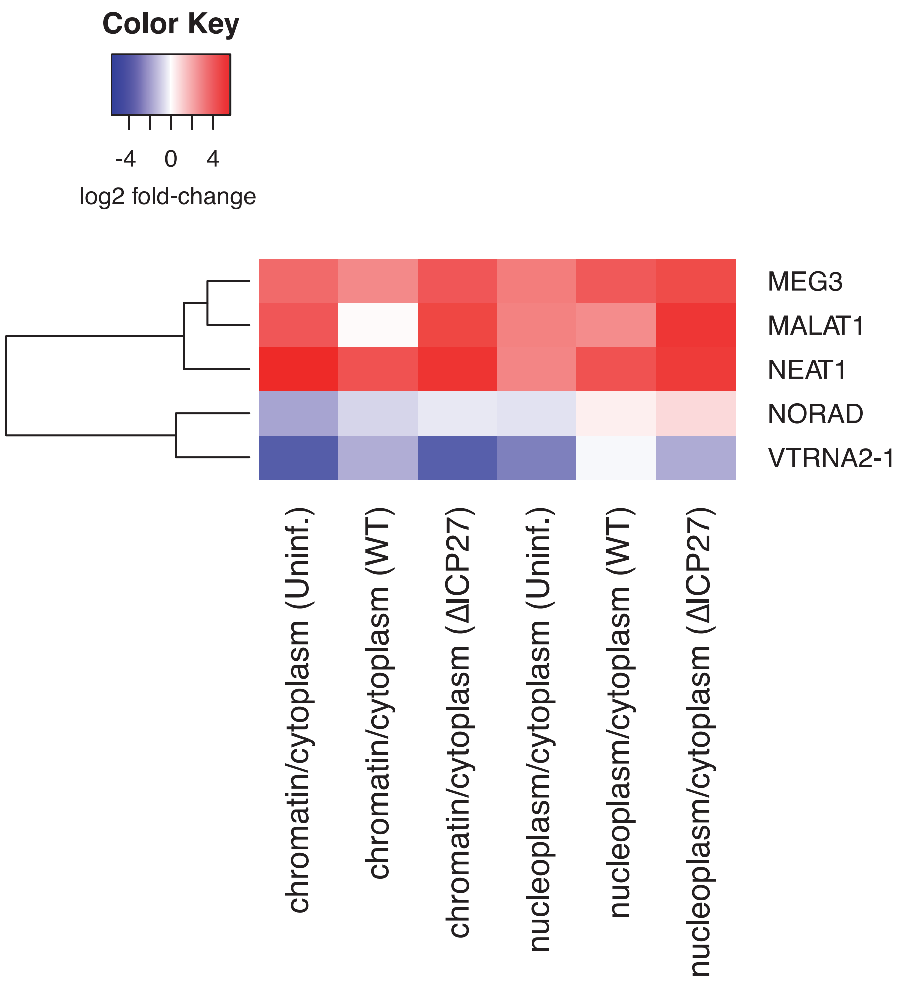

Supplementary Figure 9: Fractionation efficiencies of subcellular RNA fractions
Enrichment or depletion levels of known nuclear or cytoplasmic RNAs, respectively in chromatine associated or nucleoplasm RNA versus cytoplasm RNA.

Supplementary Tables

	million reads per dataset				
	cRNA-seq	dRNA-seq	4sU-seq	total RNA-seq	RNA from subcellular fractions
total sequenced reads	264	353	744	345	391.4
total mapped reads	31.6	227.9	259.1	270.3	332
total mapped reads (human)	25.6	227.2	198	260.1	261.6
total mapped reads (HSV1)	6	0.7	61.1	10.2	70.4
uniquely mapped reads	22.6	140	224.6	218.7	317.4
uniquely mapped reads (human)	16.6	139.3	165.5	209.1	253.2
uniquely mapped reads (HSV1)	6	0.7	59.1	9.6	64.2

Supplementary Table 1. Mapping statistics

Description of read yield, total and uniquely mapped reads for all the sequencing datasets used.

Supplementary Data

Supplementary Data 1. HSV-1 transcripts

List of all identified Transcripts including their location, name, gene cluster, and information about which dataset or method identified its TiSS.

Supplementary Data 2. HSV-1 splicing events

List of all possible splicing events with the corresponding read numbers spanning a splice-junction and not spanning it at the 5'- and 3'-end for 4sU-seq, total RNA, RNA from subcellular fraction in wild-type and ΔICP27 mutant and from data from Tang et al (*4*). Furthermore, the type indicates if it was already known, if it was identified by PacBio sequencing or by Illumina sequencing. Rows marked in green indicate the splice-junctions that were included into the final annotation.

Supplementary Data 3. List of all HSV-1

List of all identified ORFs. All ORFs of the previous reference annotation are labeled as CDS (coding sequence).

Supplementary Data 4. HSV-1 ORF function and localization prediction

Information about the function, localization and GO terms of identified ORFs.

Supplementary Data 5. Truncated HSV-1 ORFs

List of truncated ORFs including information about their location, name, and start and stop codons used.

Supplementary Data 6. HSV-1 ORFs with N-terminal extensions (NTEs)

List of ORFs with N-terminal extensions (NTEs) initiating from non-canonical start codons.

Supplementary Data 7. Validation of HSV-1 ORFs by mass spectrometry

List of ORFs identified by mass spectrometry including the number of peptides per ORF (HFF=human foreskin fibroblast data, HLF=human lung fibroblast data; All=any peptide within the protein/polypeptide are counted, Novel=only peptides outside of previously known proteins are counted).

Supplementary Data 8. HSV-1 orphan ORFs

List of ORFs for which no obvious corresponding transcript initiating within 500 nt upstream was identified.

Supplementary Data 9. Primers and gene synthesis constructs

Primers and gene synthesis constructs used.

Supplementary Methods

Code availability

Our tool iTiSS (integrative Transcriptional Start Site caller) and scripts used to validate it and create the Figures and Tables and analyze the omics data are available at zenodo (doi: https://doi.org/10.5281/zenodo.2621226).

cRNA-seq

Total RNA was isolated from infected cells using TRIzol and subjected to Ribo-zero rRNA depletion according to manufacturer's instructions. Equal volumes of 2x Alkaline Fragmentation Solution (2 mM EDTA,10 mM Na_2CO_3, 90 mM $NaHCO_3$, pH 9.3) were added to samples and incubated at 95°C 20min, followed by ethanol precipitation and gel purification of 50-80nt fragments on a 15% TBE-Urea gel. RNA was extracted from the gel by overnight incubation in RNA Gel Extraction Buffer (300 mM sodium acetate pH 5.5, 1 mM EDTA, 0.25% SDS), precipitated with isopropanol and resuspended in 10µL 10mM Tris pH 8.0. 2'/3' phosphates were removed with T4 PNK before ligation with the L3-App adapter (*10*), followed by reverse transcription with the SuperScript III First-Strand Synthesis System

(ThermoFisher #18080051), cDNA circularization with CircLigase II (EpiCentre), and BamHI digestion as described in the iCLIP protocol before library amplification with the AccuPrime SuperMix (ThermoFisher #12344040) and library sequencing as described in the main text.

TiSS profiling

Definitions:

- Reads: As this analysis is about transcriptional start sites, only the 5' ends of reads are considered. Consequently, inside this "TiSS profiling" section the term "read" refers to the very 5' end of that specific read.
- Potential TiSS: A single nucleotide position inside the genome, that fulfills at least one of the nine criteria mentioned below.
- TiSS: A transcriptional start site found in our final annotation (*bona fide* TiSS), which refers to a single nucleotide position with a +/- 5 bp window around it (i.e., two potential TiSS at positions 100 and 105 are merged into a single TiSS).
- Dataset: With at least two biological replicates available for all experiments included in this study, each replicate was tested individually for each respective criterion. A criterion was only considered to be fulfilled if it was fulfilled in both replicates.

Each position on both strands of the HSV-1 genome is tested for each of the following 9 criteria:

(i) Significant accumulation of dRNA-seq reads thereby defining a TiSS

(ii) Significant accumulation of cRNA-seq reads thereby defining a TiSS

(iii) Significantly stronger transcriptional activity downstream than upstream of the potential TiSS in the cRNA-seq dataset

(iv) Significant temporal changes (compared to upstream/downstream regions) in potential TiSS read levels during the course of infection in the cRNA-seq dataset

(v) A TiSS called in the MinION dataset with a maximum distance of 20 nt downstream.
For this purpose, the transcripts provided in the Supplementary Table of Depledge et al. (*2*) were used.

(vi) A TiSS called in the PacBio data in close proximity (+/- 5 nt).
For this purpose, we manually corrected the GFF-file provided alongside the GEO-submission for the PacBio data (*1*), which was inconsistent with the transcripts reported in the paper.

(vii) Significant differences of temporal changes upstream compared to downstream of the potential TiSS in read levels during the course of infection in the 4sU-seq dataset.

(viii) Significantly stronger transcriptional activity downstream than upstream of the potential TiSS in the 4sU-seq dataset.

(ix) The presence of an ORF at most 250 bp downstream which was not yet explained by another transcript.

In the following, the rationale and details of each criterion will be discussed. For criteria ii, iii and viii the reads were pooled over all time points of the respective time course data for each position.

i. **dRNA-seq read accumulation**:
In the dRNA-seq dataset, almost all reads map to the actual transcription start site while only a minor fraction of reads map to transcript bodies (~300-fold enrichment of TiSS). Indeed, less than 2% of positions in the HSV-1 genome have reads. However, the number of reads per potential TiSS varied substantially and there appeared to be some regions in the HSV-1 genome that produced increased noise levels, which we needed to account for. Consequently, a moving window approach (+/-100 bp around the currently assessed position) was chosen to identify potential TiSS and compare read levels to the surrounding environment. Large differences indicate the presence of a potential TiSS. Manual inspection of the data indicated that a maximum of three viral potential transcripts initiated within a 201 bp window around a *bona fide* TiSS. Consequently, at each step, iTiSS sorts the read counts inside the window in descending order and compares the read count of the currently observed position with the fourth highest. If the fold-change between them exceeded a threshold of four, the position was scored as a potential TiSS. Furthermore, within a +/-5 nt window of many cellular and viral TiSS, we observed additional accumulations of reads. These presumably reflect polymerases that initiate transcription a few base-pairs upstream or downstream of the dominant TiSS. Sequencing bias may exaggerate these alternative TiSS. iTiSS accounts for this by removing all read counts in the window that are in proximity (+/-5 bp) of the currently observed position. Additionally, to prevent division by zero, a pseudo-count of 1 read count was added to each position.

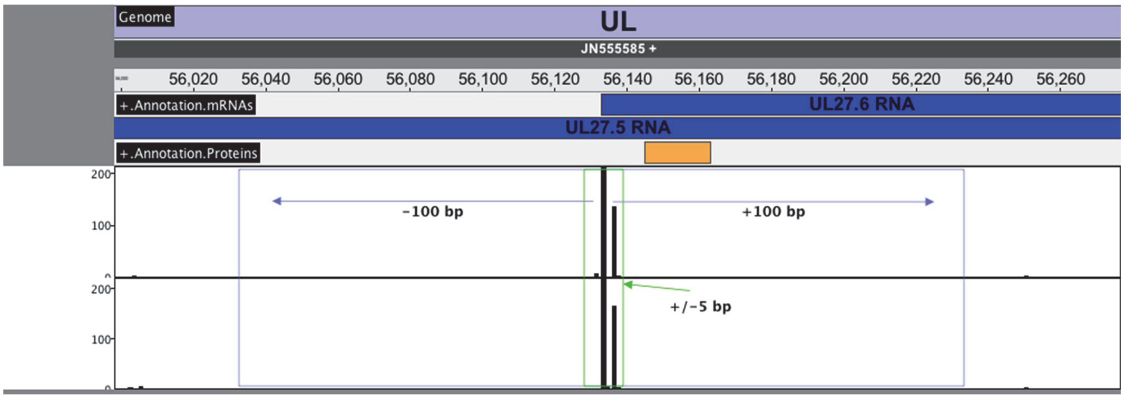

ii. **cRNA-seq read accumulation**: While the cRNA-seq dataset also shows a strong enrichment of reads at transcription start sites (~18-fold enrichment of TiSS), it contains a lot more reads that map to the gene body in comparison to the dRNA-seq data. Thereby, cRNA-seq data not only depict the TiSS but also visualize the full-length transcripts that initiate from them. The total numbers of reads at the TiSS and within the gene body varied widely akin to the dRNA-seq data set. For this reason, we again chose a moving window approach. To account for reads within the gene body, three moving windows (101 bp each) were used. The first window is located 100 bp downstream of the currently observed position, the second 100 bp upstream and the third +/- 50 bp around it. In HSV-1, multiple transcripts commonly use the same poly(A)-site. Consequently, many transcriptional start sites are located inside another transcript, which started further upstream. Let *a* and *b* be two transcriptional start

sites that both utilize the same poly(A)-site with *a* being located upstream of *b*. With cRNA-seq reads mapping throughout the whole transcript, we would expect the read counts of *b* to be greater than the read levels in between *a* and *b* as well as downstream of *b*. iTiSS accounts for this by employing a widely used outlier filtering approach. The interquartile range, i.e. the difference between read counts of the position at the third quartile and the read counts of the position at the second quartile is calculated for all three windows. Next, the difference between the currently observed position and the third quartile in each window is calculated. If this exceeds a threshold of 5 times the interquartile range within all windows, the position was considered to be a potential TiSS. The third window is used as an additional filter to prevent calling potential TiSS in noisy areas of the genome. Those areas usually comprise a significant number of reads accumulating in a ~100 bp region with no reads before and after it. Consequently, around 50% of the first and second window would contain positions with no reads mapping to them, moving the second and third quartile in those windows closer to zero and therefore falsely increasing the number of called potential TiSS. Additionally, if less than 50% of positions in all three windows contained reads mapping to them, the region was disregarded and the respective reads considered to reflect experimental noise.

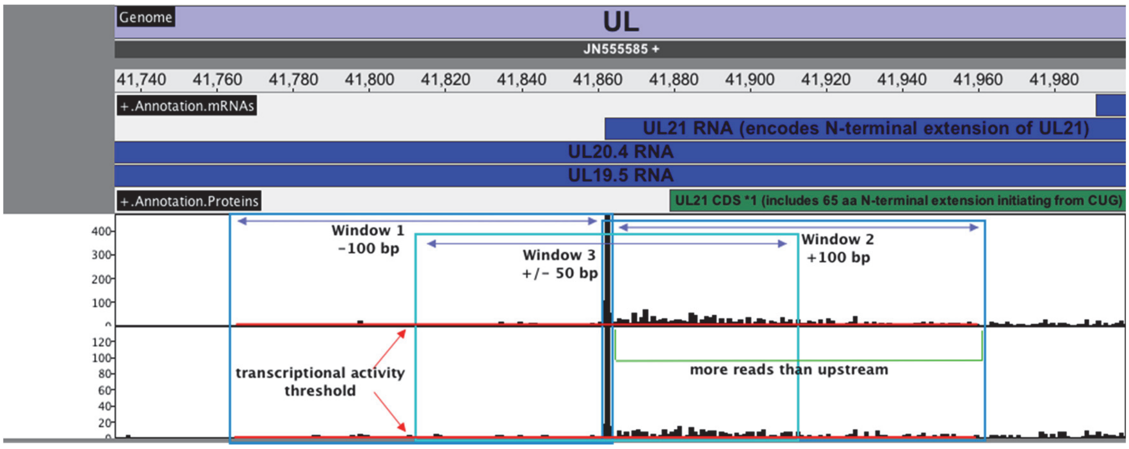

iii. **cRNA-seq transcriptional activity**: Due to reads mapping throughout the whole transcript, cRNA-seq offers another possibility for validating potential TiSS. Again, let *a* and *b* be two transcriptional start sites with the same poly(A)-site, and *a* being located upstream of *b*. We expect the read levels downstream of *b* to be greater than the read levels between *a* and *b*, as read levels downstream of *b* originate from two transcripts. By contrast, the read levels between *a* and *b* only originate from one transcript. iTiSS accounts for that by comparing the read levels 100 bp upstream against the read levels 100 bp downstream using a Fisher's exact test. To this end, a threshold is defined by the mean of the read levels from the upstream window. Afterwards, the number of positions with more or less reads mapping to them than the threshold for the upstream and downstream window, respectively, are put into a contingency table. To prevent calling noise, the number of positions to look at is reduced by only validating positions that were called by (ii) but with a lower interquartile range threshold of three. The p-value threshold was set to 1% and afterwards corrected for multiple testing using the Benjamini-Hochberg correction.

iv. **cRNA-seq kinetic activity**: Our cRNA-seq dataset comprises five different time points (0, 1, 2, 4 and 8 h p.i.). Transcription of most viral genes becomes detectable as early as 2 h p.i. However, some TiSS are predominantly utilized at a certain stage (or time) of infection (e.g. true late genes). Peaks in cRNA-seq/dRNA-seq are an indicator of a TiSS, or could e.g. result from cloning bias. Cloning bias should be reproducible across all time points. Thus, it can be excluded if a peak is only seen at defined time points. To test this, iTiSS performs a Dirichlet likelihood ratio test: If a potential TiSS is equally observed at all the time points, the counts follow a multinomial distribution parametrized by the read frequencies around it (+/- 20 bp). Conversely, the frequencies follow a Dirichlet distribution parameterized by the TiSS read counts (null model). If a potential TiSS is observed at only some of the time points, the multinomial parameters are fit by maximum likelihood. The p value is computed by a χ^2 test with two degrees of freedom (only 2h, 4h and 8h are considered). As in (iii), this was done for all positions that were called a potential TiSS in (ii) with the lowered threshold of three times the interquartile range. The criterion is fulfilled if the adjusted p-value (Benjamini-Hochberg) was lower than 1%.

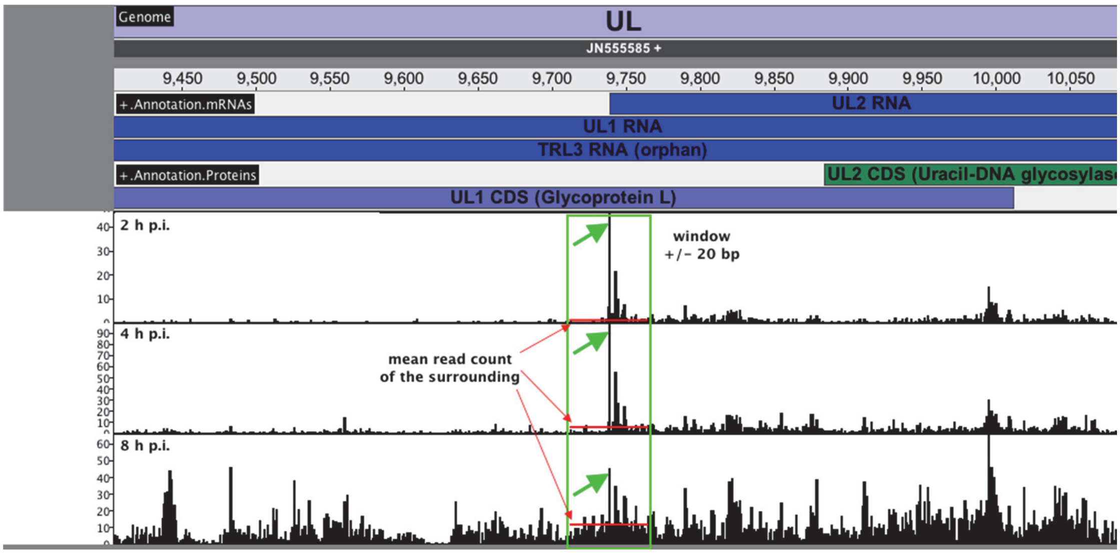

v. **MinION TiSS**: Depledge et al already called transcripts from their MinION data. However, we observed that their transcripts usually started around 15 bp downstream of the potential TiSS that we called by cRNA-seq, dRNA-seq as well as the transcripts called by PacBio from Tombacz et al. This is a common observation for MinION datasets caused by 5' degradation. Therefore, we used their transcriptional start sites as a criterion for a potential TiSS found by (i), (ii), (iii), (iv) or (vi), if Depledge et al called a transcript starting in window of up to 20 bp downstream.

vi. **PacBio TiSS**: Similar to the MinION dataset, called transcripts were already published for the PacBio data set. We used these as an additional criterion for potential TiSS found by (i), (ii), (iii), (iv) or (v) if a transcriptional start site was called by Tombacz et al in their PacBio dataset in a window of +/- 5 bp.

vii. **4sU-seq kinetics**: Similar to (iv), a region downstream of a potential TiSS that has different expression behavior over time as compared to the region upstream is an indicator of a *bona fide* TiSS. The expression behavior can be analyzed using our

previously published 4sU-seq data. First, the number of reads with a 5' end in between two potential TiSS was determined for each of the 4sU-seq samples. For two subsequent potential TiSS of the same gene locus, distinct kinetic behavior was then tested using a likelihood ratio test: Two linear models were constructed with the $\log_2$ ratio of the two corresponding read counts as dependent variable, and either no independent variable (offset only; model 1), or the time after infection of the corresponding samples as independent variable (model 2). Statistical significance was then determined using the χ^2 distribution based on the likelihood ratio of these two nested models. If the $\log_2$ ratio changes during infection, the model 2 better fits the data than the model 1. This criterion is fulfilled if the (Benjamini-Hochberg adjusted) p value was below 1%.

viii. **4sU-seq coverage:** Similar to (iii), an increase of the 4sU-seq read coverage in any sample downstream of a potential TiSS compared to upstream of it is an indicator of a *bona fide* TiSS. First, the effective length of each range in between two subsequent potential TiSS of the same gene locus was determined based on the fragment length distribution from the experiment (paired-end) and actual length of the range. The effective length of the range between TiSS *a* and *b* is a measure of the expected number of RNA fragments that can be sequenced and can originate from a transcript starting at *a* but not from a transcript starting at *b*. If the transcription termination site (TTS) is far away with respect to the fragment length distribution, the effective length equals the actual length. Closer TTS restrict the number of possible RNA fragments and the effective length is reduced accordingly. The effective length and the read count from (vii) were then used to compute a sample-specific coverage for each pair of subsequent potential TiSS. This criterion was fulfilled if the TiSS was associated with at least a 2-fold increase in coverage for at least 4 different samples.

ix. **Orphan ORF TiSS**: Every ORF needs a transcript from which it is translated. Commonly, translation initiates with the first 250 bp of an mRNA. Thus, this criterion is fulfilled if a yet unexplained ORF initiated within the next 250 bp downstream of a potential TiSS. If only one of the previous criteria is met, but an ORF is found starting downstream of it, it is more likely that the potential TiSS is correct. Please note that we carefully assessed all potential TiSS, which only fulfilled one other criterion than ix.

iTiSS validation

iTiSS implements criterion i-iv as described above. A thorough validation in terms of sensitivity and precision is difficult due to the lack of a gold-standard data set in particular for viral TiSS. To approximate sensitivity and precision, we considered annotated TiSS of cellular genes (Ensembl v90, FANTOM5 phase 1(*5*)), and used iTISS to predict those from our data.

To estimate precision, we ordered each TiSS predicted by iTiSS either in the cRNA-seq, or dRNA-seq data alone or in both of them by their enrichment factor (number of reads starting at the TiSS divided by the mean number of TiSS 20 bp upstream). We considered a TiSS to be correct, if there was an annotated TiSS in the currently annotated human transcriptome (+/-5 bp) or a peak called in the FANTOM5 dataset (+/-5bp).

For sensitivity, we ordered the protein-coding human genes in the 4sU-seq data (mock) by their TPM-values. We considered a gene to be identified, if an annotated TiSS (Ensembl v90 or FANTOM5) was predicted by iTiSS for one of its transcripts within a +/-5 bp window.

We observed a positive predictive value of iTiSS looking at the dRNA-seq or cRNA-seq dataset alone of 64.5% (1803/2796 TiSS) and 54.1% (1021/1889 TiSS), respectively. Only

looking at the TiSS observed in both datasets increased the precision to 80.2% (392/489 TiSS).

Sensitivity was 85.5% and 94.2% for cRNA-seq and dRNA-seq, respectively (71.0% for their combination), for genes with a TPM >100. The sensitivity is rapidly declining for genes expressed at lower levels. This indicates that we may have missed TiSS of weakly expressed viral mRNAs, or that the TiSS annotation (Ensembl/FANTOM5) of weakly expressed genes is not as reliable as for strongly expressed genes. However, we would like to note that many viral genes are expressed at very high levels. In comparison, weakly expressed viral transcripts, which may have been missed by iTiSS, are thus of very low abundance and much less likely to be of important functional relevance in our experimental model.

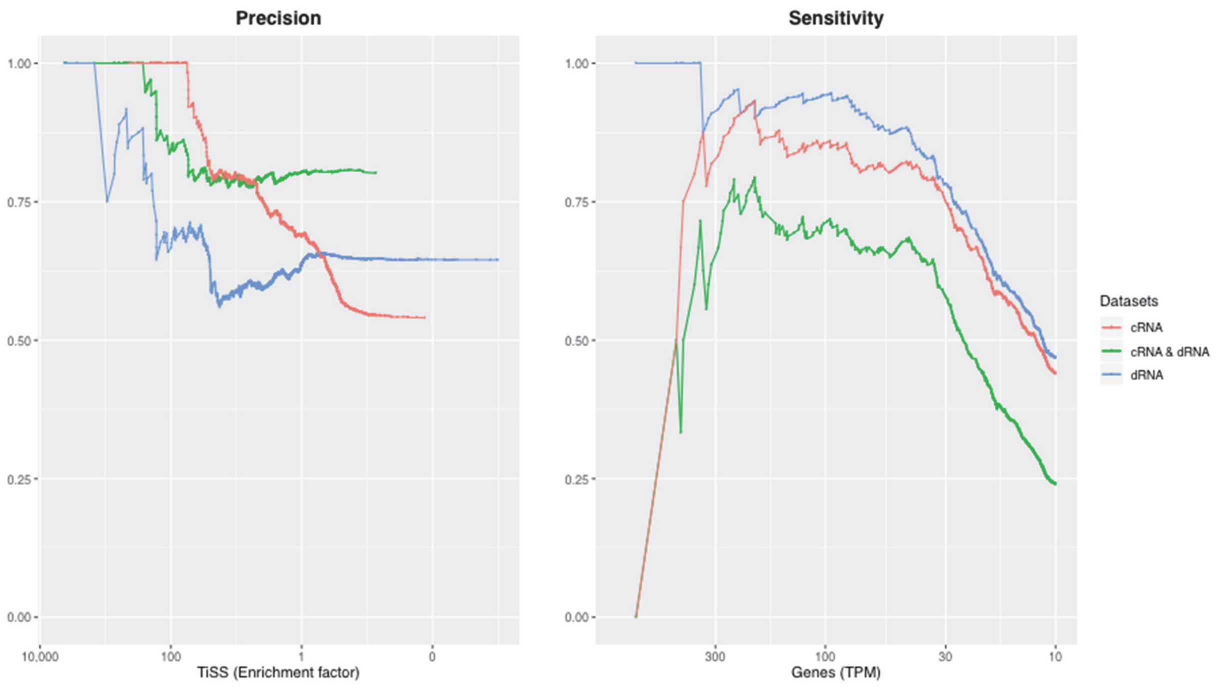

Manual curation

Once the automatic scoring was done, potential TiSS with a score $\geq$3 were accepted into our final annotation following manual inspection. Furthermore, potential TiSS with a score of 2 scored by criterion (i)-(viii) were also considered *bona fide* TiSS. Nevertheless, they were all carefully inspected manually and all found to highly likely represent *bona fide* TiSS.

All remaining potential TiSS were manually curated by looking at the data in our viewer. In particular, we consider the orphan ORF TiSS criterion (ix) the weakest piece of evidence for a *bona fide* TiSS. For this reason, we removed TiSS that only fulfilled this and one other criterion, and only kept those that exhibited additional strong evidence (for instance a fold-change between 3 and 4 instead of the picked threshold of 4 in dRNA-seq). In addition, we had a close look at the nucleotide sequence at the TiSS looking for factors that could have impeded cloning or mapping of the respective reads, e.g. poly(C) or poly(G) stretches as well as repeat regions.

Information on the reasons for including each respective potential TiSS into the final HSV-1 genome annotation are included in Suppl. Data. 1, column U.

Finally, *bona fide* TiSS were automatically extended to the next poly(A)-site. Those were manually checked for potential poly(A)-read-through transcripts that were validated by our, PacBio or MinION data. The resulting transcripts were included into our final HSV-1 genome annotation. For heavily spliced genomic loci, we also considered the PacBio and MinION data to annotate specific transcript isoforms.

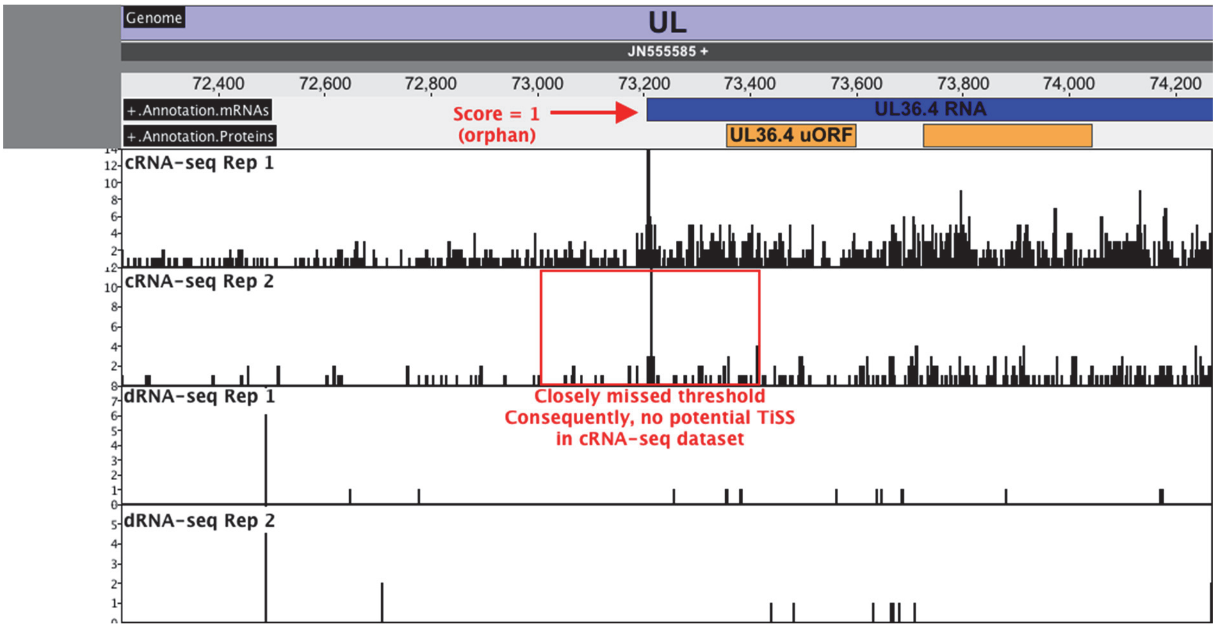

As shown in the example above, the TiSS of the UL36.4 mRNA in the cRNA-seq data was only picked up by iTiSS in Rep.1 as it just missed the threshold in Rep. 2. However, in addition to the peak at the TiSS in both replicates, the number of reads downstream of the potential TiSS around 73,200 is clearly higher than upstream of it for both replicates. Furthermore, it explains translation of UL36.4 uORF. Therefore, we included this TiSS into our final genome annotation.

ORF calling

To identify translated open reading frames from the ribosome profiling data, we used our in-house tool PRICE (*11*). Briefly, PRICE first uses an expectation-maximization (EM) algorithm to map ribosome footprints to P site codons (by respecting the probabilistic nature of RNase cleavage). It then assembles codons identified in any available sample to ORF candidates. Next, start codon candidates are identified using machine learning, incorporating information available from samples treated with translation initiation inhibitors (Harringtonine, Lactimidomycin). Finally, assembled ORF candidates are tested for a signature of active translation (more in-frame codons than out-of-frame and flanking codons) using a generalized binomial test. Here, we ran PRICE independently for each of the two replicates, and called all ORFs which were independently identified in both biological replicates. The remaining candidates were manually curated upon inspection of the data in our genome viewer (adding additional ORFs that were called in one replicate, but only slightly missed exceeding the threshold for ORF calling in the other replicate). Finally, we identify additional ORFs that had been missed by the automated PRICE approach by manually checking regions in our genome browser where our Ribo-seq data suggests ORFs but due to repetitive sequences genuine ribosome footprints were prevented from being mapped there. This, e.g. resulted in the identification of the novel spliced ORF RL2A. In this case, PRICE had initially only identified the N-terminal part (initially termed RL2 uoORF) as a stop codon, which is located in frame within the first triplet of the intron. However, as this intron is spliced out and removed, translation can continue within the downstream exon.

Mass Spectrometry

Processing of HSV-1-infected WI-38 human lung fibroblasts

After pooling labelled and unlabelled cell lysates, protein disulfide bridges were reduced in 2mM DTT for 30 minutes at 25 °C and successively free cysteines were alkylated in 11 mM iodoacetamide for 20 minutes at room temperature in the dark. LysC digestion was performed by adding LysC (Wako) in a ratio 1:40 (w/w) to the sample and incubating it for 18 hours under gentle shaking at 30 °C. After LysC digestion, the samples were diluted 3 times with 50 mM ammonium bicarbonate solution before addition of 10 µL immobilized trypsin (Applied Biosystems) were added and incubated for 4 hours under rotation at 30 °C. Digestion was stopped by acidification with 10 µL of trifluoroacetic acid (TFA) and removal of trypsin beads by centrifugation. The resulting peptide mixtures were loaded on Empore cartridges (3M) following the instructions of the manufacturer and eluted with 70% acetonitrile. The acetonitrile was then removed by evaporation in a rotation vacuum concentrator (RVC 2-33 CDplus, Christ, Germany). The samples were then fractionated by in-solution isoelectric focusing as previously described (*12*). Briefly, samples were diluted to 2.5 mL with MilliQ water, 150 µL of ampholite solution (pH range 3-10, 40% w/w, Bio-Rad) were added and the sample was then loaded into the focusing chamber of the Microrotofor device. Focusing was performed by application constant power current (1W, limiting voltage 500V, limiting current 10 mA). After reaching stable voltage (~ 2.5 hours) the focusing was allowed to run for other 30 minutes before harvesting. The resulting fractions were desalted on STAGE Tips (max 15 µg per StageTip), dried and reconstituted to 25 µL of 0.5 % acetic acid in water (*13*).

Five microliters were injected in duplicate on a LC-MS/MS system (NanoLC-Ultra [Eksigent] coupled to LTQ-Orbitrap Velos [Thermo]), using a 240 minutes gradient ranging from 5% to 40% of solvent B (80% acetonitrile, 0.1 % formic acid; solvent A= 5 % acetonitrile, 0.1 % formic acid). For the chromatographic separation, 20 cm long capillary (75 µm inner diameter) was packed with 1.8 µm C18 beads (Reprosil-AQ, Dr. Maisch). One end of the capillary nanospray tip was generated using a laser puller (P-2000 Laser Based Micropipette

Puller, Sutter Instruments), allowing fretless packing. The nanospray source was operated with a spray voltage of 2.1 kV and ion transfer tube temperature of 260 °C. Data were acquired in data dependent mode, with one survey MS scan in the Orbitrap mass analyzer (resolution 60000 at m\z 400) followed by up to 20 MS\MS (LTQ-Orbitrap Velos) in the ion trap on the most intense ions (intensity threshold = 500 counts). Once selected for fragmentation, ions were excluded from further selection for 30 seconds, in order to increase new sequencing events.

Processing of HSV-1-infected primary human foreskin fibroblasts

Cells were harvested at the indicated time points, washed with ice-cold PBS, snap-frozen in liquid nitrogen and stored at -80 °C prior to filter-aided sample preparation (FASP; *14, 15*). Cell pellets were lysed in 4% SDS/100 mM Tris HCl pH 7.4 supplemented with cOmplete protease inhibitor cocktail (Roche), and sonicated at 4 °C using a Diagenode Bioruptor. Protein concentrations were determined using the Pierce BCA Protein Assay kit (Thermo Scientific) and light, medium and heavy lysates combined 1:1:1 by protein mass (total 45 µg protein/replicate). Lysates were transferred to Microcon-30 kDa centrifugal filter units (Millipore), reduced (100 mM DTT) and alkylated (50 mM iodoacetamide) at room temperature, washed with a total of 5 column volumes of 8 M urea/100 mM Tris HCl pH 8.5, then digested with 1 µg modified sequencing grade trypsin (Promega) in 50 mM ammonimum bicarbonate at 37 °C for 12 hrs. Peptide eluates were collected by centrifugation and stored at -80 °C prior to fractionation.

HpRP was conducted using a Dionex UltiMate 3000 UHPLC system (Thermo Scientific) powered by an ICS-3000 SP pump with an Agilent ZORBAX Extend-C18 column (4.6 mm x 250 mm, 5 µm particle size). Peptides were resolved using a linear 40 min 0.1% - 40% acetonitrile gradient at pH 10.5 with eluting peptides collected in 15 s fractions. Peptide-rich fractions were concatenated across the gradient to give 10 pooled fractions/sample, dried using an Eppendorf Concentrator then re-suspended in 15 µL mass spectrometry solvent (3 % acetonitrile, 0.1 % TFA).

Mass spectrometry data were generated using an Orbitrap Fusion Tribrid mass spectrometer (Thermo Scientific). Peptides were fractionated using an RSLCnano 3000 (Thermo Scientific) with solvent A comprising 0.1% formic acid and solvent B comprising 80% acetonitrile, 20% H_2O, 0.1% formic acid. Peptides were loaded onto a 50 cm Acclaim PepMap C18 column (Thermo Scientific) and eluted using a gradient rising from 3 to 40% solvent B by 73 min at a flow rate of 250 nL/min. MS data were acquired in the Orbitrap at 120,000 fwhm between 350–1500 m/z. Spectra were acquired in profile with AGC 5×10^5. Ions with a charge state between 2+ and 7+ were isolated for fragmentation in top speed mode using the quadrupole with a 1.6 m/z isolation window. HCD fragmentation was performed at 33% collision energy with fragments detected in the ion trap between 350–1400 m/z. AGC was set to 5×10^3 and MS2 spectra were acquired in centroid mode.

Principles of the new nomenclature of HSV-1 transcripts and ORFs

1. No previously annotated ORFs were renamed to avoid causing confusions with previous work. All viral ORFs and transcript mentioned in the 6th Edition of Fields of Virology were included.
2. To differentiate all new ORFs from the previously reported ORFs, we labeled all previous ORFs as "coding sequences", e.g. UL1 CDS.
3. We differentiate long (≥100aa; named "ORF") from short (3 – 99aa) viral ORFs.
4. We differentiated five different kinds of sORFs. These include upstream open reading frames ("uORFs"), upstream overlapping ORFs ("uoORFs"), internal ORFs ("iORFs") and downstream ORFs ("dORFs"). In addition, sORFs, which are expressed from transcripts not containing any large ORF were named "sORFs", e.g. UL34.5 sORF 1 and 2.
5. Translation of "uORFs" both starts and terminates upstream of a large ORF. A transcript can have multiple uORFs (e.g. UL14 uORF 1 and 2). In case a transcript does not encode any ORF >100aa, all short ORFs it encodes are labeled "sORFs", e.g. UL30.5 sORF 1 and UL30.5 sORF 2.
6. In contrast to uORFs, uoORFs overlap with the main ORF expressed from the respective transcript.
7. Internal ORFs (iORFs) are located within the coding sequence of large ORFs but expressed in a different frame. In principle, two scenarios can explain their translation.
   (i) They can be translated by ribosomes, which have missed the TaSS of the main ORF (e.g. UL20 iORF) and thus initiate translation at the iORF.
   (ii) They can result from alternative independent transcripts initiating downstream of the respective TaSS of the main ORF, e.g. UL53 iORF RNA #2.
   iORFs were thus not labeled as "orphan".
8. Finally, a small number of downstream ORFs (dORFs) were annotated. These represent sORFs located downstream of large ORFs, which could not be explained by an independent transcript, e.g. UL39.6 dORF 1 and 2 downstream of UL39.6 ORF. Their translation may result from ribosomes re-initiating after completing the translation of the large ORF located further upstream. Therefore, they were not labeled as "orphan". However, in most cases it is equally likely that they are translated from yet unidentified viral transcripts.
9. In principle, novel viral transcripts, ORFs and sORFs can all result in the introduction of a new viral gene identifier, e.g. UL28.5.
   a. Any novel large viral ORF, e.g. UL36.5 ORF, was given a new identifier unless it was overlapping with another large ORF. In the rare case that two overlapping viral ORFs (translated from different frames) were obviously expressed from the same transcript, these were named A and B, e.g. UL40.7A ORF and UL40.7B ORF as well as TRL2 CDS and TRL2A ORF.
   b. For viral transcripts to be given a new identifier, this required a transcription start site (TiSS) >500 nucleotides upstream of the closest other transcript, e.g. UL54.5 RNA (orphan).
   c. Any sORF >20aa in length that could not be attributed to another viral gene as a either uORF, uoORF, iORF or dORF was given a new identifier, e.g. UL27.5 sORF 1.
10. Numbering of new identifiers was defined based on the location of the TiSS or TaSS in relation to the neighboring previously annotated genes (x and x+1) on either strand. In case multiple new identifiers were required between two annotated genes, the most strongly expressed gene was named x.5, the neighboring ones x.4 and x.6. As annotations of additional genes by previous studies did not all follow the same rules in

regards to neighboring genes, we tried to choose the best possible numbering for each locus.

11. Usage of alternative transcription start sites is a very common phenomenon in the HSV-1 genome. Many of the additional transcriptions contain additional uORFs and thereby explain their expression. As such, we commonly observed >1 distinct TiSS within a window of 250 nt up- or downstream of the transcript of a given locus. The main TiSS was defined by the highest cRNA-seq or dRNA-seq peak. Within a window of +/-10 nt, no additional TiSS were annotated. TiSS identified by cRNA-seq, dRNA-seq and PacBio commonly matched perfectly at single nucleotide level.
12. Any transcript that did not contain an ORF within its first 500 nucleotides (nt) was labeled as “orphan”, e.g. UL54.5 RNA (orphan).
13. Any ORF or sORF for which no transcript could be identified that explained its translation within the transcript’s first 500 nt was labeled as “orphan”, e.g. US11.5 ORF (orphan).
14. Additional transcripts initiating upstream of the main transcript were labeled “*”+”number” with higher numbers reflecting increasing distance to the main TiSS, e.g. UL24 RNA *1.
15. Transcripts initiating downstream of the main transcript were labeled “#”+”number” with higher numbers reflecting increasing distance to the TiSS of the main transcript, e.g. UL41 RNA #1 and UL41 RNA #2.
16. Transcript experiencing alternative splicing were labeled as “iso1, iso2…”.
17. The annotation of uORFs was based on the most prominent transcript of the respective locus, e.g. UL6 uORF. Alternative TiSS commonly explained the expression of additional uORFs, e.g. UL6 RNA *1 explained UL6 uORF RNA *1.
18. Transcripts with retained introns were labeled as “i”+”number”, e.g. IRL2 RNA i1. The respective ORF variants were labeled accordingly, e.g. IRL2 ORF RNA i1
19. N-terminal extensions of ORFs were labeled with “*1”, e.g. UL50 CDS *1. In case of a second, longer N-terminal extension this was labeled “*2”, e.g. UL50 CDS *2. All N-terminal extensions of previously identified proteins initiated from non-AUG start codons. Both the start codon and the length of the extension are indicated in brackets, e.g. US3 CDS *1 (includes 23 aa N-terminal extension initiating from CUG).
20. N-terminal truncations of ORFs were labeled with “#”+”number”, e.g. UL37.6 ORF #1. We did not observe any more than 1 truncated version of a given ORF.
21. ORFs, sORFs and transcripts expressed from the repeat regions of the viral genome where named accordingly, e.g. IRL2.5 ORF and TRL2.5 ORF. We did not differentiate the three other possible orientations of the unique long and unique short regions.

Annotations in the vicinity of the latency-associated transcript:

We were unable to detect the full-length LAT transcript in our data from lytically infected HFF but annotated it nevertheless. However, translation of the two previously described viral ORFs, ORF-O and ORF-P was readily detectable. In addition, we identified the corresponding RNA (ORF-O/P RNA). Both ORFs and the respective transcript were only apparent at early but not late times of infection. This is consistent with its reported repression by ICP4. However, our data indicate that ORF-O does not result from a frameshift in ORF-P but rather initiates from an ACG start codon 76nt upstream of the start codon of ORF-P.

Data sets

The following data sets were included in the analysis as depicted in Fig. 1a. This information is also found in tabular form in the Source-Data file.

1) Total RNA-seq data from mock, 2, 4, 6 and 8 h p.i. (n=2)
2) 4sU-seq data obtained in hourly intervals during the first 8 h of infection (n=2)

These data were published in (*6*). In brief, primary human fibroblasts were infected at an MOI of 10. Newly transcribed RNA was labeled for 1h by adding 500 µM 4sU to the cell culture medium prior to cell lysis. Newly transcribed RNA was purified as described. The corresponding total RNA samples were analyzed every second hour of infection.

Accession: GSE59717

3) cRNA-seq samples from mock, 1, 2, 4 and 8 h p.i. (n=2)

cRNA-seq generates sequencing libraries from total cellular RNA using a cloning protocol which is based on 3'-adaptor ligation and circularization. It was initially described by Stern-Ginossar et al (*7*). The cloning protocol was initially developed for ribosome profiling but was subsequently found to result in a substantial enrichment of transcript 5'-ends due to the circularization step. We decided to name this approach "cRNA-seq" to differentiate it from the second TiSS profiling approach termed dRNA-seq (differential RNA-seq) (*8*).

Primary human fibroblasts were infected with wild-type HSV-1 at an MOI of 10. Total RNA was isolated at the indicated time of infection and subjected to cRNA-seq.

Accession: GSE128324

4) dRNA-seq samples from mock and 8 h p.i. (n=2)

Primary human fibroblasts were infected with wild-type HSV-1 at an MOI of 10. dRNA-seq libraries were prepared from uninfected cells and at 8 h p.i. Library preparation was performed as described (*8*). Specificity of the approach is enhanced by treating the RNA sample with the 5'-exonuclease Xrn1. Libraries prepared from water-(H2O)-treated samples (prepared for both samples of mock and 8 h p.i.) were sequenced as controls but also show a strong enrichment of 5'-ends.

Accession: GSE128324

5) Subcellular RNA fractions from mock, wild-type HSV-1 (8h p.i.), ΔICP27 (8h p.i.) infection (n=2)

Subcellular RNA fractions were prepared as described (*9*). Total, cytoplasmic, nucleoplasmic and chromatin-associated RNA were prepared and subjected to RNA-seq following rRNA depletion. The data from mock and wild-type HSV-1 infection have already been published (*9*). The ΔICP27 samples were prepared in the same experiment as the mock and wild-type HSV-1 infection and were not included in the primary publication.

Accession: GSE128880

6) Third generation sequencing

The data obtained using PacBio (*1*) and MinION (*2*) platforms were reanalyzed. For details on the experimental setup please see the respective papers.

Accession: PRJEB27861 (MinION), GSE97785 (PacBio)

7) Ribo-seq analysis from mock, 1, 2, 4, 6 and 8 h p.i. (n=2)
8) Ribo-seq + Harringtonine from mock, 2 and 8 h p.i. (n=2)
9) Ribo-seq + Lactimidomycin from mock, 2 and 8 h p.i. (n=2)

The data of the standard Ribo-seq analysis without chemical inhibitors to enrich for TaSS have already been published (*6*). In brief, primary human fibroblasts were infected with wild-type HSV-1 at an MOI of 10. Cells were harvested at the indicated time points by snap-freezing and subsequent lysis in a buffer containing cycloheximide to prevent ribosomes from continuing with translation.

TaSS using Harringtonine and Lactimidomycin were prepared in the same experiments as the standard Ribo-seq samples. Here, Harringtonine or Lactimidomycin were added to the cell culture medium 30 min prior to cell lysis. Ribo-seq libraries were prepared as described (*6*).

Accession: GSE60040 (Ribo-seq), GSE128324 (TaSS profiling)

10) SILAC whole proteome mass spec analysis (MS-Cambridge)

Primary human fibroblasts comprehensively labeled with heavy, medium or light lysine and arginine were infected with wild-type HSV-1 at an MOI of 10. All three condition (heavy, medium and light) were utilized for all three infection conditions (mock, 4 and 8 h p.i.) providing us with three replicates of triple-SILAC whole proteome mass spec data.

Accession: PXD013010, PXD013407 (PRIDE)

References:

1. D. Tombácz, Z. Csabai, A. Szűcs, Z. Balázs, N. Moldován, D. Sharon, M. Snyder, Z. Boldogkői, Long-Read Isoform Sequencing Reveals a Hidden Complexity of the Transcriptional Landscape of Herpes Simplex Virus Type 1. *Front Microbiol*. **8**, 1079 (2017).
2. D. P. Depledge, K. P. Srinivas, T. Sadaoka, D. Bready, Y. Mori, D. G. Placantonakis, I. Mohr, A. C. Wilson, Direct RNA sequencing on nanopore arrays redefines the transcriptional complexity of a viral pathogen. *Nat. Commun.* **10**, 754 (2019).
3. C. M. Calton, J. A. Randall, M. W. Adkins, B. W. Banfield, The Pseudorabies Virus Serine/Threonine Kinase Us3 Contains Mitochondrial, Nuclear and Membrane Localization Signals. *Virus Genes*. **29**, 131–145 (2004).
4. S. Tang, A. Patel, P. R. Krause, Hidden regulation of herpes simplex virus 1 pre-mRNA splicing and polyadenylation by virally encoded immediate early gene ICP27. *PLOS Pathog.* **15**, e1007884 (2019).
5. A. R. R. Forrest, H. Kawaji, *et al*. A promoter-level mammalian expression atlas. *Nature*. **507**, 462–470 (2014).
6. A. J. Rutkowski, F. Erhard, A. L'Hernault, T. Bonfert, M. Schilhabel, C. Crump, P. Rosenstiel, S. Efstathiou, R. Zimmer, C. C. Friedel, L. Dölken, Widespread disruption of host transcription termination in HSV-1 infection. *Nat. Commun.* **6**, 7126 (2015).
7. N. Stern-Ginossar, B. Weisburd, A. Michalski, T. K. L. Vu, M. Y. Hein, S. X. Huang, M. Ma, B. Shen, S. B. Qian, H. Hengel, M. Mann, N. T. Ingolia, J. S. Weissman, Decoding Human Cytomegalovirus. *Science*. **338**, 1088–1093 (2012).
8. C. M. Sharma, J. Vogel, Differential RNA-seq: the approach behind and the biological insight gained. *Curr. Opin. Microbiol.* **19**, 97–105 (2014).
9. T. Hennig, M. Michalski, A. J. Rutkowski, L. Djakovic, A. W. Whisnant, M.-S. Friedl, B. A. Jha, M. A. P. Baptista, A. L'Hernault, F. Erhard, L. Dölken, C. C. Friedel, HSV-1-induced disruption of transcription termination resembles a cellular stress response but selectively increases chromatin accessibility downstream of genes. *PLOS Pathog.* **14**, e1006954 (2018).
10. I. Huppertz, J. Attig, A. D'Ambrogio, L.E. Easton, C.R. Sibley, Y. Sugimoto, M. Tajnik, J. König, J. Ule. iCLIP: protein-RNA interactions at nucleotide resolution. *Methods*. **65**(3): 274-287 (2014).
11. F. Erhard, A. Halenius, C. Zimmermann, A. L'Hernault, D.J. Kowalewski, M.P. Weekes, S. Stefanovic, R. Zimmer, L. Dölken. Improved Ribo-seq enables identification of cryptic translation events. *Nat. Methods.* **15**, 363–366 (2018).
12. C. Adamidi, Y. Wang, D. Gruen, G. Mastrobuoni, X. You, D. Tolle, M. Dodt, S.D. Mackowiak, A. Gogol-Doering, P. Oenal, A. Rybak, E. Ross, A. Sanchez Alvarado, S. Kempa, C. Dieterich, N. Rajewsky, W. Chen. *De novo* assembly and validation of planaria transcriptome by massive parallel sequencing and shotgun proteomics. *Genome Res*. **21**(7), 1193-200 (2011).
13. J. Rappsilber, Y. Ishihama, M. Mann. Stop and go extraction tips for matrix-assisted laser desorption/ionization, nanoelectrospray, and LC/MS sample pretreatment in proteomics. *Anal. Chem*. **75**(3), 663-70 (2003).
14. E.J.D. Greenwood, N.J. Matheson, K. Wals, D.J.H. van den Boomen, R. Antrobus, J.C. Williamson, P.J. Lehner. Temporal proteomic analysis of HIV infection reveals remodelling of the host phosphoproteome by lentiviral Vif variants. *eLife.* **5**, e18296 (2016).
15. J.R. Wiśniewski, A. Zougman, N. Nagaraj, M. Mann. Universal sample preparation method for proteome analysis. *Nat. Methods.* **6**, 359-62 (2009).
