# Supplementary material for: Integrative functional genomics decodes herpes simplex virus 1
Source: Nat Commun. 2020 Apr 27;11:2038. doi: 10.1038/s41467-020-15992-5 (PMC7184758; doi:10.1038/s41467-020-15992-5)
Supplement: Supplementary file 3 — Reporting Summary [file 41467_2020_15992_MOESM3_ESM.pdf]

nature research

Corresponding author(s): Florian Erhard, Lars Dölken

Last updated by author(s): Mar 31, 2020

Reporting Summary

Nature Research wishes to improve the reproducibility of the work that we publish. This form provides structure for consistency and transparency in reporting. For further information on Nature Research policies, see Authors & Referees and the Editorial Policy Checklist .

Statistics

For all statistical analyses, confirm that the following items are present in the figure legend, table legend, main text, or Methods section.

n/a	Confirmed	
☐	☒	The exact sample size (*n*) for each experimental group/condition, given as a discrete number and unit of measurement
☐	☒	A statement on whether measurements were taken from distinct samples or whether the same sample was measured repeatedly
☐	☒	The statistical test(s) used AND whether they are one- or two-sided<br>*Only common tests should be described solely by name; describe more complex techniques in the Methods section.*
☐	☒	A description of all covariates tested
☐	☒	A description of any assumptions or corrections, such as tests of normality and adjustment for multiple comparisons
☐	☒	A full description of the statistical parameters including central tendency (e.g. means) or other basic estimates (e.g. regression coefficient) AND variation (e.g. standard deviation) or associated estimates of uncertainty (e.g. confidence intervals)
☐	☒	For null hypothesis testing, the test statistic (e.g. *F*, *t*, *r*) with confidence intervals, effect sizes, degrees of freedom and *P* value noted<br>*Give P values as exact values whenever suitable.*
☐	☒	For Bayesian analysis, information on the choice of priors and Markov chain Monte Carlo settings
☐	☒	For hierarchical and complex designs, identification of the appropriate level for tests and full reporting of outcomes
☒	☐	Estimates of effect sizes (e.g. Cohen's *d*, Pearson's *r*), indicating how they were calculated

Our web collection on statistics for biologists contains articles on many of the points above.

Software and code

Policy information about availability of computer code

Data collection	ImageStudioLite (LI-COR), ZEISS ZEN Microscopy software (Zeiss), cellSense (Olympus)
Data analysis	Gedi 1.0.2 (https://github.com/erhard-lab/gedi)<br>Bowtie 1.0<br>iTiSS 1.0 (https://doi.org/10.5281/zenodo.2621226)<br>PRICE 1.0.1 (https://github.com/erhard-lab/gedi)<br>MaxQuant 1.6.5.0<br>lfc R package (0.2.1)<br>Fiji<br>The following additional R packages: ComplexHeatmap, circlize, ggseqlogo, ggplot2, reshape2, plyr, scales, ggforce, ggrepel and gridExtra

For manuscripts utilizing custom algorithms or software that are central to the research but not yet described in published literature, software must be made available to editors/reviewers. We strongly encourage code deposition in a community repository (e.g. GitHub). See the Nature Research guidelines for submitting code & software for further information.

Data

Policy information about availability of data

All manuscripts must include a data availability statement. This statement should provide the following information, where applicable:

- Accession codes, unique identifiers, or web links for publicly available datasets
- A list of figures that have associated raw data
- A description of any restrictions on data availability

All sequencing data have been deposited in the Gene Expression Omnibus (GEO) database with accession codes GSE128324 (Translation start site profiling, transcription start site profiling), GSE59717 (4sU-seq and total RNA-seq), GSE60040 (ribosome profiling) and GSE128880 (cytoplasmic, nucleoplasmic and

chromatin-associated RNA). We only used the transcripts predicted in the PacBio and MinION dataset, respectively. However, the raw data can by found at the GEO database with accession code GSE97785 (PacBio), and from the European Nucleotide Archive (ENA) with the study accession code PRJEB27861 (MinION). The mass spectrometry proteomics data have been deposited to the ProteomeXchange Consortium via the PRIDE [1] partner repository with the dataset identifiers PXD013010 and PXD013407.
The following figures have associated raw data: Fig. 2,3,4,6.
All raw data are fully available.

Field-specific reporting

Please select the one below that is the best fit for your research. If you are not sure, read the appropriate sections before making your selection.

- [x] Life sciences
- [ ] Behavioural & social sciences
- [ ] Ecological, evolutionary & environmental sciences

For a reference copy of the document with all sections, see nature.com/documents/nr-reporting-summary-flat.pdf

Life sciences study design

All studies must disclose on these points even when the disclosure is negative.

Sample size	*Describe how sample size was determined, detailing any statistical methods used to predetermine sample size OR if no sample-size calculation was performed, describe how sample sizes were chosen and provide a rationale for why these sample sizes are sufficient.*
Data exclusions	No data were excluded from the analysis.
Replication	All data were confirmed by at least two biological replicates. No data were excluded from the analysis.
Randomization	*Describe how samples/organisms/participants were allocated into experimental groups. If allocation was not random, describe how covariates were controlled OR if this is not relevant to your study, explain why.*
Blinding	*Describe whether the investigators were blinded to group allocation during data collection and/or analysis. If blinding was not possible, describe why OR explain why blinding was not relevant to your study.*

Reporting for specific materials, systems and methods

We require information from authors about some types of materials, experimental systems and methods used in many studies. Here, indicate whether each material, system or method listed is relevant to your study. If you are not sure if a list item applies to your research, read the appropriate section before selecting a response.

Materials & experimental systems

n/a	Involved in the study	
[ ]	[x]	Antibodies
[ ]	[x]	Eukaryotic cell lines
[x]	[ ]	Palaeontology
[x]	[ ]	Animals and other organisms
[x]	[ ]	Human research participants
[x]	[ ]	Clinical data

Methods

n/a	Involved in the study	
[x]	[ ]	ChIP-seq
[x]	[ ]	Flow cytometry
[x]	[ ]	MRI-based neuroimaging

Antibodies

Antibodies used	α-FLAG M2 (Sigma #F1804), α-β-actin C4 antibody (Santa Cruz Biotechnology #sc-47778), IRDye® 800CW goat α-mouse IgG (LI-COR #926-32210), α-mouse IgG (whole molecule)-peroxidase (Sigma #9044), α-ICP0 clone 5H7 (Santa Cruz Biotechnology #sc-56985), α-ICP27 clone H1113 (Abcam #ab53480), IRDye® 680RD goat α-mouse IgG (LI-COR #926-68070), α-mouse IgG (whole molecule)-peroxidase (Sigma #9044), Anti-FLAG antibody (GenScript #A00187), anti-mouse IgG, Alexa Fluor 488 (ThermoFisher #A11017)
Validation	No additional validation statements were provided by the manufacturers

Eukaryotic cell lines

Policy information about cell lines

Cell line source(s)	All Omics data were generated using primary human foreskin fibroblasts obtained from ECACC and cultured in DMEM with 10% FBS Mycoplex and 1% penicillin/streptomycin. 293T and WI-38 cells were obtained from ATCC. BHK-21 cells were a kind gift from Stacey Efstathiou, Cambridge, UK. Vero 2-2 cells were generously provided by Roz Sandri-Goldin, UC Irvine.

Authentication	Cells did not undergo additional validation after receiving them from the supplier.
Mycoplasma contamination	Cells and all virus stocks continuously tested negative for Mycoplasma contamination by PCR.
Commonly misidentified lines<br>(See ICLAC register)	*Name any commonly misidentified cell lines used in the study and provide a rationale for their use.*
