# Supplementary material for: Integrative functional genomics decodes herpes simplex virus 1
Source: Nat Commun. 2020 Apr 27;11:2038. doi: 10.1038/s41467-020-15992-5 (PMC7184758; doi:10.1038/s41467-020-15992-5)
Supplement: Supplementary file 4 — Description of Additional Supplementary Files [file 41467_2020_15992_MOESM4_ESM.pdf]

Description of Additional Supplementary Files

File Name: Supplementary Data 1
Description: **HSV-1 transcripts**
List of all identified Transcripts including their location, name, gene cluster, and information about which dataset or method identified its TiSS.

File Name: Supplementary Data 2
Description: **HSV-1 splicing events**
List of all possible splicing events with the corresponding read numbers spanning a splicejunction and not spanning it at the 5'- and 3'-end for 4sU-seq, total RNA, RNA from subcellular fraction in wild-type and ΔICP27 mutant and from data from Tang et al (4). Furthermore, the type indicates if it was already known, if it was identified by PacBio sequencing or by Illumina sequencing. Rows marked in green indicate the splice-junctions that were included into the final annotation.

File Name: Supplementary Data 3
Description: **HSV-1 ORFs / Predicted function / Mass Spectrometry validation**
(A) List of all identified ORFs. All ORFs of the previous reference annotation are labeled as CDS (coding sequence). (B) Information about the function, localization and GO terms of identified ORFs. (C) List of ORFs identified by mass spectrometry including the number of peptides per ORF (HFF=human foreskin fibroblast data, HLF=human lung fibroblast data; All=any peptide within the protein/polypeptide are counted, Novel=only peptides outside of previously known proteins are counted).

File Name: Supplementary Data 4
Description: **Truncated HSV-1 ORFs**
List of truncated ORFs including information about their location, name, and start and stop codons used.

File Name: Supplementary Data 5
Description: **HSV-1 ORFs with N-terminal extensions (NTEs)**
List of ORFs with N-terminal extensions (NTEs) initiating from non-canonical start codons.

File Name: Supplementary Data 6
Description: **HSV-1 orphan ORFs**
List of ORFs for which no obvious corresponding transcript initiating within 500 nt upstream was identified.

File Name: Supplementary Data 7
Description: **Primers and gene synthesis constructs**
Primers and gene synthesis constructs used.

File Name: Supplementary Data 8
Description: **HSV-1 orphan ORFs**
List of ORFs for which no obvious corresponding transcript initiating within 500 nt upstream was identified.

File Name: Supplementary Data 9
Description: **Primers and gene synthesis constructs**
Primers and gene synthesis constructs used.
